# Impact in Participatory Health Research

**DOI:** 10.1155/2018/3907127

**Published:** 2018-10-21

**Authors:** Michael T. Wright, Jon Salsberg, Susanne Hartung

**Affiliations:** ^1^Institute for Social Health, Catholic University of Applied Sciences Berlin, Köpenicker Allee 39-57, 10318 Berlin, Germany; ^2^Graduate Entry Medical School, University of Limerick, Limerick V94 T9PX, Ireland; ^3^Department of Health, Nursing, and Management, Neubrandenburg University of Applied Sciences, Brodaer Straße 2, 17033 Neubrandenburg, Germany

The idea for this special issue arose from the first* International Scientific Meeting on the Impact of Participatory Health Research* organized by the International Collaboration for Participatory Health Research (ICPHR), the German Network for Participatory Health Research (PartNet), the Institute of Population and Public Health, Canadian Institutes of Health Research (CIHR), and Community-Based Research Canada (CBRC). The conference took place in June 2015 at the Center for Interdisciplinary Research (ZiF) in Bielefeld, Germany. Experts in PHR from eleven countries met to launch an international discussion on what impact means in the participatory research process, how to maximize the impact of the research, and how to observe and document what impact has occurred. Several of the themes discussed at the conference are addressed in this issue.

Participatory health research (PHR) is an approach in which those people whose life or work is the subject of the research influence the research process. PHR enables nonacademic researchers to take part directly in deciding the topic of the research, the research questions, the mode of data collection, the interpretation of the results, and the dissemination of the findings. Through this involvement, PHR seeks not only to describe and explain health problems and their causes, but also to bring about the necessary social change for the benefit of people's health. In PHR learning and research are not considered separate entities. Social learning (learning together and from each other) is a fundamental dimension of the PHR process, and the continual cycle of “look, reflect, act” underpins the dynamics of developing a connected knowing [1]. This means trying to understand the other person or idea through dialogue from relations of trust and empathy [2]. Everyone learns as a coresearcher to differing degrees. Ideally, the process should engage the participants in transformative learning, i.e., changes in the way they see the world and themselves [3, 4], through interactive processes which address both the personal and the collective. In turn, this generates an intention of being able to act based on the research findings, thus having a wider impact beyond the scientific community. On the whole, how social change is defined is largely determined by whether the approach is pragmatic (that is, focused on issues of practical utilization) or emancipatory (where the focus is on changing the way people think and act in their world)—or an attempted combination of both [5, 6].

The work of Cook et al. [7] has demonstrated the difficulty authors have in recognizing and articulating impact in PHR. This includes recognizing the impact of participation on the research process and capturing the longitudinal aspect of impacts that occur long after a project has been completed. In an extensive review of the English language literature, Jagosh et al. [8] identified, selected, and appraised a large-variety sample of primary studies describing PHR partnerships. They used key realist review concepts to analyze and synthesize the data, employing the PHR partnership as the main unit of analysis (compare Jagosh et al. [9]). The link between the participatory research process and the outcomes in these partnerships was explained using the middle-range theory of* partnership synergy*, which demonstrates how PHR can (1) ensure culturally and logistically appropriate research; (2) enhance recruitment capacity; (3) generate professional capacity and competence in stakeholder groups; (4) result in productive conflicts followed by useful negotiation; (5) increase the quality of outputs and outcomes over time; (6) increase the sustainability of project goals beyond funded time frames and during gaps in external funding; and (7) create system changes and new unanticipated projects and activities.

A review by Staley [10] describes positive and negative impacts which can result from PHR, based on an extensive review of published and gray literature on the INVOLVE strategy for public involvement in the research of the National Health Service in the UK. This includes impact on the research process (agenda, design, delivery, and ethics), impact on the public involved, impact on academic researchers, impact on other research participants, impact on the wider community, impact on community organizations, and impact on change processes (e.g., improved service delivery).

In this special issue the topic of impact in PHR is examined from several perspectives. Janet Harris and colleagues call attention to the lack of clarity in the literature regarding what participation means and the frequent lack of information on participatory processes. Taking into account these and other challenges, they make recommendations for conducting systematic reviews of impact in PHR, citing recent reviews while providing practical examples for dealing with issues at each stage of the review process. In the article by Erica Di Ruggiero and Nancy Edwards another overarching issue regarding impact in PHR is addressed; namely, the interface between implementation research and PHR. They compare and contrast these two relatively recent developments in research practice, highlighting the ways in which PHR can contribute to the impact of health research, more generally. John G. Oetzel and colleagues present an empirically-tested model for examining the various forms of impact in PHR as an integrated part of research planning and evaluation. Their model is the result of a large-scale study in the United States involving PHR practitioners which has since been applied to many different settings.

The remaining articles in this issue are examples of how three very diverse projects in three different countries have addressed the issue of impact. Lisa Gibbs and colleagues present a case study of* Beyond Bushfires*, a large, multisite, mixed method study of the psychosocial impacts of major bushfires in Victoria, Australia. The challenges of balancing local interests with state-wide implications are explored in the description of the methods of engagement and the study processes and outcomes. A similar tension between local and national interests is described by Michael T. Wright and colleagues in their article on* PartKommPlus—German Research Consortium for Healthy Communities*. The consortium, composed of seven subprojects focused on participation in local strategies of health promotion, has struggled to bring together the knowledge arising from the wide variety of contexts and methods. The contribution of Sónia Dias and colleagues provides a well-documented example of how PHR can impact a specific health issue in a specific community, with both social and political implications. A participatory HIV research project was conducted with sex workers and men who have sex with men to understand epidemiological HIV dynamics and associated sociobehavioural factors in Portugal. Advantages of the participatory process were encountered but also challenges, evidencing the dynamic and complex nature of each project stage.

The collection of articles in this special issue inspires and challenges us to examine the complex and important issue of impact in the emerging field of participatory approaches to health research.
